# Growth rate of ascending thoracic aortic aneurysms in a non-referral-based population

**DOI:** 10.1186/s13019-022-01761-6

**Published:** 2022-02-02

**Authors:** Gabe Weininger, Makoto Mori, Sameh Yousef, David J. Hur, Roland Assi, Arnar Geirsson, Prashanth Vallabhajosyula

**Affiliations:** 1 Section of Cardiac Surgery, Yale University School of Medicine, 330 Cedar Street, Boardman Building 204L, New Haven, CT 06520 USA; 2 Section of Cardiovascular Medicine, Yale School of Medicine, New Haven, CT USA

**Keywords:** Ascending aortic aneurysm, Growth rate, Referrel based population, Computed tomography

## Abstract

**Background:**

Prior studies on ascending thoracic aortic aneurysm (ATAA) growth rates have reported approximately 1 mm of growth per year but these studies are based on referral-based study populations which are biased towards the highest risk patients who may not represent the true natural history of aortic aneurysm disease. We aimed to characterize the growth rate of ATAAs in a non-referral-based population, using a large institutional database of computed tomography (CT) scans.

**Methods:**

We queried the 21,325 CT scans performed at our institution between 2013 and 2016 on patients ages 50–85 years old for radiologic diagnosis of aortic aneurysm or dilatation. 560 patients were identified to have aortic dilatation > 4 cm, of which 207 had follow-up scan intervals > 6 months. This comprised our non-referral-based study population. Linearized annual aneurysm growth rates were calculated by dividing the change in aortic size by the time interval between CT scans.

**Results:**

The median time interval between scans was 2.7 years (interquartile range [IQR] 1.5–4.2) for the 207 patients included in the study. The median initial aneurysm size was 4.3 cm (IQR 4.1–4.5). 38.2% (n = 79) of patients did not experience aortic dilatation. The median growth rate was 0.13 mm/year (IQR − 0.24 to 0.49). Of patients in the top quartile of growth rates, 26.9% of patients were female whereas 12.9% of patients were female in the bottom three quartiles of growth rates.

**Conclusion:**

While some patients’ ATAAs may grow at previously published rates of around 1 mm/year, this is not the predominant pattern in a non-referral-based population and may over-estimate the overall growth rate of ATAAs.

## Introduction

The mostly asymptomatic nature of ascending thoracic aortic aneurysms (ATAAs) require characterization of the growth rate of ATAAs to inform the appropriate timing of surgical intervention and surveillance practices [1]. Prior studies on the ATAA growth rate have reported approximately 1 mm of growth per year but these studies are limited by small sample sizes, mixed imaging modalities and measurement techniques, and are based on referral-based study populations [2]. Studies using referral-based study populations may be biased by the highest risk patients [2, 3] and the findings may not represent the true natural history of aortic aneurysm disease. We aimed to characterize the rate of ATAA growth in a non-referral-based population, using a large institutional database of computed tomography (CT) scans.

## Methods

We queried the 21,325 CT scans performed at our institution between 2013 and 2016 on patients ages 50–85 for radiologic diagnosis of aortic aneurysm or dilatation. 560 patients were identified to have aortic dilatation > 4 cm, of which 207 had follow-up scan intervals > 6 months. This comprised our non-referral-based study population.

**Fig. 1:**
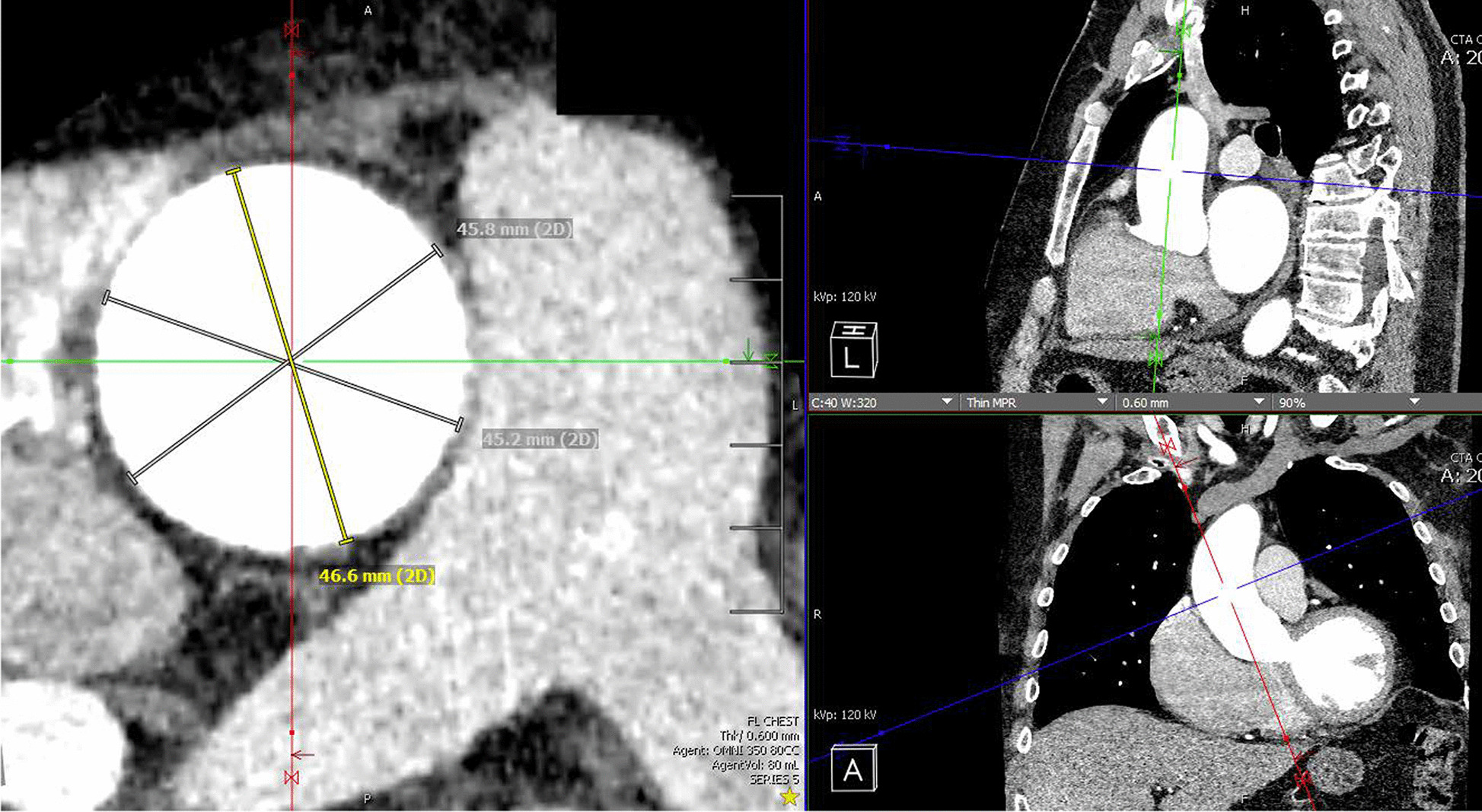
Double oblique measurement of the ascending thoracic aorta. The figure shows the measurement of the ascending aorta using the double oblique technique. Maximum transverse diameter was measured on multiplanar reconstruction images in planes perpendicular to the aortic wall, from outer-to-outer edge. Final measurements were calculated as an average of 3 diameters measured at 60-degrees apart from each other

The double oblique technique was used to measure the maximum transverse diameter of the ascending aorta, from outer-to-outer edge (Visage 7, Visage Imaging, Inc., San Diego, CA). Final measurements were calculated as an average of 3 diameters measured 60-degrees apart from each other (Fig. 1). Linearized annual growth rate was calculated by dividing the change in aortic size by the time interval between CT scans.

Patient characteristics were compared between those with faster and slower growth rates using Wilcoxon ranked sum test for continuous variables and chi-squared tests for categorical variables. Faster growth rates were defined as those in the top 25 percentile of growth rates in our study cohort. Continuous variables were summarized with median and interquartile range (IQR) with statistical significance defined as *p* < 0.05.

The Institutional Review Board at Yale University approved this study and individual consent was waived (IRB 2000027551). The study followed the Strengthening the Reporting of Observational Studies in Epidemiology (STROBE) reporting guidelines for cohort studies.

## Results

The median time interval between scans was 2.7 years (interquartile range [IQR] 1.5–4.2) for the 207 patients included in the study. The median growth rate was 0.13 mm/year (IQR − 0.24 to 0.49). The median initial aneurysm size was 4.3 cm (IQR 4.1–4.5). 38.2% (n = 79) of patients did not experience aortic dilatation. Of patients in the top quartile of growth rates (median 0.95 mm/year IQR 0.68–1.63), 26.9% of patients were female whereas 12.9% of patients were female in the bottom three quartiles of growth rates (Table 1).

**Table 1:** Patient characteristics

Patient characteristics	Combined (n = 207)	Growers (n = 52)	Non-growers (n = 155)	*p* Value
Initial aneurysm size (cm) Median [IQR]	4.3 [4.1–4.5]	4.3 [4.1–4.5]	4.3 [4.2–4.5]	0.99
Growth rate (mm/yr)	0.133 [− 0.24–0.49]	0.95 [0.68–1.63]	− 0.03 [− 0.4–0.2]	** < 0.001**
Age	74 [67,80]	74 [67,81]	74 [67–80]	0.66
BMI	27.1 [24.3–31.2]	27.2 [23.6–31.4]	27 [24.3–31.4]	0.82
Sex (% female)	34 (16.4%)	14 (26.9%)	20 (12.9%)	**0.03**
Hypertension	151 (72.9%)	41 (78.9%)	110 (71%)	0.35
COPD	38 (18.3%)	9 (17.3%)	29 (18.7%)	0.98
Previous MI	17 (8.2%)	6 (11.5%)	11 (7.1%)	0.47
Family history	37 (17.8%)	8 (15.4%)	29 (18.7%)	0.73
Smoker	10 (48.3%)	24 (46.2%)	76 (49%)	0.84
Aortic valve disease	21 (10.1%)	6 (11.5%)	15 (9.7%)	0.91
Diabetes	37 (17.9%)	11 (21.2%)	26 (16.8%)	0.61
CHF	31 (15%)	11 (21.2%)	20 (12.9%)	0.22

Bold indicates *p* < .05

*BMI* body mass index, *COPD* chronic obstructive pulmonary disease, *CHF* congestive heart failure, *MI* myocardial infarction

The table summarizes the patient characteristics of the 207 patients who were identified to have ascending aortic dilatation > 4 cm on computed tomography scans with follow-up intervals > 6 months. Growers were defined as those in the top 25 percentile of growth rates in our study cohort. Characteristics were compared between the growers and non-growers using Wilcoxon ranked sum test for continuous variables and chi-squared tests for categorical variables

## Discussion

Our results suggest that while some patients’ ATAAs may grow at previously published rates of around 1 mm/year, this is not the predominant pattern and may over-estimate the overall growth rate of ATAAs. Referral-based study populations are often comprised of higher-risk patients who are more often symptomatic and experience aortic complications, including rupture and dissection, at higher rates [1, 4]. This may contribute to an overestimation of the overall ATAA growth rate in the existing body of literature, which has placed the rate around 1 mm/year [2, 3] in contrast to our finding of 0.13 mm/year with only a subset of patients growing at ≥ 1 mm/year. Other likely sources of overestimation include the use of axial aortic diameter measurements instead of the double oblique measurement technique [5] and combining ascending and descending thoracic aneurysms in the same study cohort [2].

Our study is limited by the use of contrast and non-contrast CT scans as well as ECG gated and non-gated scans; though these factors are not consistently accounted for in other studies on ATAA growth rates, they could influence aortic diameter measurements.

## Abbreviations

ATAA: Ascending thoracic aortic aneurysm
CT: Computed tomography
IQR: Interquartile range
ECG: Electrocardiogram
